# Studies in Wound Infections: On the Question of Bacterial Symbiosis in Wound Infections

**Published:** 1917-12

**Authors:** 


***Studies in Wound Infections : On the Question of Bacterial Symbiosis in Wound Infections.*** By S. R. Douglas, Capt. I. M. S. (Retired) and A. Fleming, and L. Colebrook; all of the bacteriological department of the Medical Research Committee, National Insurance Act, and of the Inoculation Department of St. Mary's Hospital. Abs. from the *Lancet*, April 21, 1917.

The authors recall the importance of bacterial symbiosis which has for many years been well established, especially in the association of anaerobes and aerobes, and of B. influenza with staphylococcus or A. xerosis.

In the wounds of the present war, bacterial flora is varied. The primary infection is usually fecal. In the early stages of a wound the anaerobic organisms, B. perfringens (aerogenes capsulatus) and organisms of putrefaction, flourish together with streptococci, B. proteus, and diphtheroids. The anaerobes gradually disappear, and finally only streptococci, staphylococci, diphtheroids, and, occasionally, B. proteus remain.

Besides the B. tetanus, the organisms causing the worst infections in the early stages of a wound are B. perfringens (aerogenes capsulatus), possibly B. malignant œdema, and streptococci, while in the later stages streptococci are responsible for almost all the serious complications. Although the others do not produce such complications, their presence in the wound may be of very great importance if it can be shown that they in any way stimulate the action of the more dangerous varieties. The experiments are reported in the paper to show that this is probably the case.

The culture medium used in the experiments was usually human serum unaltered or treated with sufficient trypsin to neutralize the antitryptic powers, which treatment Wright has shown to be essential in rendering the fluids of the wound more suitable for the growth of bacteria.

In *milk* it was found that the broth culture of B. perfringens would grow only when transferred in the dilutions of 1 : 10 or 1 : 100. When, however, it was combined with staphylococcus and streptococcus, it flourished in sowings up to 1 : 1,000,000.

In *serum* untreated with trypsin B. perfringens did not flourish when sowed in any proportion, but it grew from sowings of dilutions from 1 : 1 to 1 : 1,000,000 when combined with diphtheroid organisms. In serum treated with trypsin it flourished alone in sowings of only 1 : 1 and 1 : 10 dilution but in combination with diphtheroids it flourished in proportions up to 1 : 1,000,000. When thus combined, B. perfringens (aerogenes capsulatus (developed gas much more rapidly than under any other conditions : it showed first in 2 1/2 hrs., and within 4 hrs. the gas equaled the bulk of the culture fluid.

In a further series of experiments with diphtheroids, it was found that 9 different strains of the diphtheroids stimulated the growth of B. perfringerns approximately to the same extent.

When B. perfringens was combined with a coliform bacillus and B. proteus, it was found that the coliforms stimulated it to more than twice its growth alone, and B. proteus increased its growth more than five-fold.

In symbiosis with streptococci and staphylococci, it was found that B. perfringens was not only stimulated itself by the presence of the other organisms, but that it contributed in a similar manner to their growth.

Further experiments with varying technique showed an immensely increased activity of B. perfringens when grown in symbiosis with staphylococci or streptococci, and that this increase of activity remained the same when the serum media were neutralized eithre with respect to their alkalinity or to their antitryptic power.

Experiments performed to ascertain the influence of streptococci in symbiosis with the *bacillus of malignant edema* showed that cultures of this bacillus would not grow when sown in dilutions of 1 : 1, but that in symbiosis with streptococci it thrived in proportions up to 1 : 1,000,000.

In similar experiments with B. Hibler as well as the bacillus of malignant edema, it was found that two strains of diphtheroids, streptococcus faecalis, and staphylococcus, (ordinary non-sporing organisms found in wounds) greatly stimulate the growth of both B. Hibler and B. of malignant edema, both of which have been said to cause gaseous gangrene.

It was ascertained also that streptococci and staphylococci are greatly stimulated in growth by the presence of B. perfringens; and that streptococci are stimulated by the presence of diphtheroids although diphtheroids appear themselves to be retarded in growth by the presence of the streptococci.

The authors also by experiment demonstrated the absorption of oxygen by various aerobes (diphtheroids, staphylococcus, streptococcus, B. pyocyaneus, and coliform bacteria); but they believe that this action is not alone responsible for the stimulus afforded to anaerobes by their presence.

With regard to the relation of symbiosis to gas gangrene, the authors conclude :

" Gas gangrene... is caused by the invasion of the tissues by certain anaerobic bacilli, the commonest being the B. perfringens. It is well known that very large doses of pure cultures of this bacillus have to be inoculated into animals in order to produce this disease with certainty; again Wright has shown that to obtain a growth of this bacillus in serum a very heavy implantation is necessary, but if the serum is altered by the neutralization of its alkalinity, or its antitryptic power, a very much smaller sowing will produce an abundant culture. From these experiments he concluded that the conditions leading to such an alteration of the blood fluids play an important part in the production of gas gangrene.

" Taking these facts into consideration, together with many of the clinical aspects of gas gangrene, it would seen apt to compare the action of B. perfringens in the production of this disease with a high explosive which is comparatively harmless until the explosive action is started by a detonator. Keeping this simile in view, it is obviously of the utmost importance to ascertain those conditions which act as " detonators " in the growth of this bacillus, and the experiments detailed above show that over and above those factors which have been described by Wright there is another — namely, symbiosis — and that this is *in vitro* experiments the most important yet described, since the growth of B. perfringens obtained in serum under such conditions is not only much more rapid in development and more copious in quantity, but also follows the lightest implantations ".

The authors believe that symbiosis may be responsible for some of the phenomena of gas gangrene generally considered baffling; such as, terrible infections from only a few bacilli, the onset of gas gangrene after careful surgical cleansing, and the "explosive" character of gas production within a few hours.

